# Chd7 regulates lipid metabolism and swim bladder inflation in zebrafish

**DOI:** 10.1016/j.jlr.2026.101062

**Published:** 2026-05-18

**Authors:** Maximilian Breuer, Gabrielle Fortier, Jaskaran Singh, Sophie Charron, Betelhem Kassa, Priyanka Jamadagni, Charlotte Zaouter, Shunmoogum A. Patten

**Affiliations:** 1 INRS – Centre Armand Frappier Santé Biotechnologie, Laval, QC, Canada; 2 Centre d'Excellence en Recherche sur les Maladies Orphelines - Fondation Courtois (CERMO-FC), Université du Québec à Montréal (UQAM), Montréal, QC, Canada

**Keywords:** Zebrafish, Dyslipidemia, Chd7, Swim bladder, lipid metabolism, CHARGE syndrome

## Abstract

The chromodomain helicase remodeling enzyme, CHD7, has been directly linked to CHARGE syndrome, however recent studies show a wide variability of pathways regulated by the chromatin remodeling enzyme. Here, we show that *chd7* mutant zebrafish present with a collection of dysregulated lipid metabolism enzymes in line with Pparγ regulated lipid metabolism. Consequently, we link lipid metabolism to swim bladder development and observe decreased size and non-inflated swim bladder during larval development. While loss-of-function of *chd7* does not appear to affect the formation of the swim bladder during early stages and early pathways, it is required for expression of lipid metabolism enzymes, subsequently resulting in failure to inflate the swim bladder. Analysis of known underlying pathways indicate a functional role of fatty acid synthesis and lipid binding. Our investigations could show that significant depletion of fatty acid enzyme, *elovl1,* and the fatty acid binding protein, *fabp7b*, parallel to *pparγ* depletion, result in a dyslipidemia and subsequent failure to inflate the swim bladder. Our study is the first to investigate the multisystemic role of *chd7* in correlation to swim bladder development and its fundamental underlying pathway in lipid metabolism, showing a necessity of *chd7* for lipid homeostasis.

The chromodomain (ATP-dependent) helicase DNA binding protein 7 (CHD7) is a key chromatin regulator involved in several functions in the developing organism. Its function is essential in regulating gene expression and has been studied focusing mostly on neural development (1). CHD7 is involved in numerous pathways including regulation of neural stem cell quiescence and proliferation, oligodendrocyte differentiation and myelination, neural crest cell formation and migration, as well as embryonic stem cell-specific gene expression (2, 3, 4, 5, 6, 7, 8, 9, 10). Recent studies, also show that Chd7 regulates adipogenic and osteogenic differentiation in mesenchymal stem cells via PPARγ (11).

Mutations in CHD7 have been directly linked to CHARGE syndrome (CS; #OMIM214800), a multisystemic congenital disorder characterized by craniofacial defects, neurological defects, heart defects and growth retardations, among others (12, 13, 14). To investigate the characteristics observed in CS, the zebrafish model organism has proven highly efficient in modelling the symptomatic and misregulated development associated with CS (15, 16, 17, 18, 19). Along these lines, one major characteristic observed in *chd7* mutants and morphants is failure to inflate the swim bladder (17, 19), which this study will investigate to detail. Swim bladders of teleosts share an evolutionary and genetic link with lung development and function in mammals (20, 21). Furthermore, respiratory involvement was early characterized as a significant portion of patients associated with CS, often due to upper airway abnormalities and choanal atresia (22, 23). Strikingly, over 40% of morbidity and mortality of early infant deaths in CHARGE syndrome have been associated with respiratory issues (23). Strikingly, transcriptomic expression profile, genetic regulation and morphology of swim bladder and lung are highly conserved (21, 24). In line with the basic anatomical structure, the underlying mechanisms in early development appear largely conserved, therefore allowing for the use of zebrafish in understanding the involvement of mechanistic pathways. Furthermore, zebrafish have proven successful in identifying therapeutic targets by small compound screenings and increasingly as a model for respiratory damage and lung injury (25, 26).

Swim bladder development is based on budding during early stages (36–65 h post fertilization (hpf)) by evagination from the gut and differentiation into the three layers of epithelium, mesenchyme and outer mesothelium; extension/elongation (65–96 hpf) and subsequently inflation (96–120 hpf) (27). Swim bladder budding, extension, and inflation have been linked to well-characterized pathways, including WNT signaling and SHH-related pathways. However, misregulation in the vascular innervation, lysosome-related organelles and fatty acid metabolism have shown to be necessary in swim bladder development and inflation (27, 28, 29, 30, 31). This regulation in swim bladder development is a crucial requirement for teleost development to ensure buoyancy, upright swimming, development, mobility, and is vital for survival.

Specifically, fatty acid and lipid synthesis show strong co-expression with the mesothelium of the swim bladder, indicating a necessity for proper lipid composition at the swim bladder creating a lipid-rich wall that allows oxygen exchange (32). Zebrafish depend on fatty acid metabolism and active transport from the yolk prior to 5 dpf, before larvae can supplement fatty acids and lipids by feeding, therefore zebrafish have proven effective in the study of lipid transport and metabolism (33, 34). Fatty acid metabolism and lipid composition, transport and synthesis are also required for proper neural development. One major 3-Ω fatty acid, docosahexaenoic acid (DHA) has shown to be required in early development, specifically for brain development, reproduction and *elovl* (elongase of very long chain fatty acids protein) gene expression (35, 36). As a matter of fact, supplementation with DHA has shown to improve growth, survival and swim bladder inflation in other marine fish such as Amberjack fish (37).

In this study we show that *chd7*^*−/−*^ mutants present with a dysregulated lipid metabolism in early development. Furthermore, we successfully connect this metabolic defect to an abnormal lipid composition in the developing larvae, which, subsequently, causes *chd7*^*−/−*^ mutants to fail to inflate the swim bladder due to depletion of key metabolic enzymes.

## Materials and Methods

### Zebrafish husbandry

Zebrafish were maintained, grown, and kept in regulations previously described (38). Fish and embryos were maintained at 28.5°C at a 12 h/12h light/dark cycle and staged as previously described (39). To inhibit pigment formation 0.003% phenylthiourea (PTU) dissolved in egg water was used at 24 hpf. *chd7*^*−/−*^ mutants were previously described (17). All experiments were performed in compliance with the guidelines of the Canadian Council for Animal Care and approved by the INRS ethics committee.

### Paraffin sections/HE staining

Zebrafish specimens were fixed in 4% paraformaldehyde and embedded in paraffin. Transverse sections (3–5 μm) of paraffin-embedded specimens were deparaffinized in xylene and were rehydrated in a graded series of ethanol. Serial sections were collected and stained with hematoxylin (STATLAB Medical Products, LLC), washed with alcohol-acid and rinsed. Then sections were soaked in saturated lithium carbonate solution and rinsed. Lastly, sections were stained with eosin Y (STATLAB Medical Products, LLC) and mounted.

### Whole mount in situ hybridization (WISH)

In situ probe generation was done using specific Primers for: *anxa5b, fabp7b, elovl1* and *pparg* with sequences summarized in supplemental Table S1. First, PCR fragments were cloned into pcrII-TOPO dual promotor (ThermoFisher) plasmid and validated by sequencing. Finally, Probes were transcribed by in vitro transcription using SP6 or T7 polymerase using the digoxigenin labelling kit (Roche). WISH was performed as previously described (40).

### TUNEL and fluorescence immunochemistry

Whole-mount TUNEL staining to determine apoptosis was performed at 5 dpf according to the manufacturer’s protocol (Roche). Whole mount fluorescence immunohistochemistry for proliferation (pH3) was done using the primary antibody pAb Rabbit Anti-phospho-Histone H3 (Ser10) (Millipore, Cat# 06–570) 1:200 and secondary (AlexaFluor488). Samples were imaged using a Zeiss LSM780.

### Oil Red O staining

Lipid staining was performed as previously described (41). In short, larvae were fixed in PFA overnight. Samples were then preincubated in 60% isopropanol for 30 min and then stained in 0.3% Oil Red O in 60% isopropanol for 3 h. Samples were then washed in PBS and imaged using an AxioZoom V16 (Zeiss).

### Compound treatment

Treatment of larvae was performed in 6-well dishes using 5 ml of E3 media and corresponding compounds. Media and compounds were replenished daily, and dead larvae were removed. Compounds used are PPAR-gamma specific antagonist Bisphenol A diglycidyl ether (BADGE) (1–10 μM) and PPAR-gamma specific agonist Rosiglitazone (RGZ) (2 μM). Compounds were dissolved in DMSO, which was also used as a vehicle control.

### RNA-Seq Analysis

Bulk RNA-Seq analysis was carried out on three independent batches of 10 *chd7*^*+/+*^ and *chd7*^*−/−*^ 2dpf larvae (whole larvae; N = 3, n = 10), corresponding to experimental triplicates. Total RNA was extracted using the RNeasy Mini kit (Qiagen) according to the manufacturer’s instructions. RNA quality control and next-generation RNA sequencing were performed at the Genomics Core Facility of the Institute for Research in Immunology and Cancer (IRIC, University of Montreal) using 2100 bioanalyzer (Agilent) and the Illumina NextSeq 500 instrument, respectively. RNA sequencing data analysis was performed by the Bioinformatics Core Facility of IRIC. About 90% of high-quality reads were mapped onto the zv9 version of the zebrafish genome using TopHat version 2.0.10. Differential gene expression analysis was assessed by the DeSeq2 package using R software. We used fgsea package in R (version 4.3.1) for the gene set enrichment analysis (GSEA). We imported gene sets from a GMT file and concurrently, a CSV file containing DEGs (*P*. adjust<0.05), was transformed into a named vector (geneList) of average log2 fold changes, sorted in descending order. Utilizing the fgsea package in R, we performed GSEA with parameters set to a minimum gene set size of 10 and a maximum of 500. The results were filtered to retain pathways with an FDR value < 0.25. To visualize, we employed the cnetplot function from the enrichplot package and visualized the top enriched pathways and their corresponding genes in a network plot. The color gradient displays the direction and magnitude of fold change: teal represents upregulated, and salmon represents downregulated.

### RT-qPCR

RT-qPCR was used to determine relative mRNA expression levels. Whole mRNA was isolated with TriReagent® (Sigma) and cDNA synthesized with SuperScript®Vilo™ kit (Invitrogen) using 1 μg of isolated RNA. RT-qPCR was run using the LightCycler® 96 (Roche) and 2X SYBR Green Master Mix (Bioline). *elf1a* was used as the reference gene for normalization and primers shown in supplemental Table S1 were used.

### BIODIPY FL C12 injections and imaging

Medium length fatty acid compound BODIPY™ FL C12 (4,4-Difluoro-5,7-Dimethyl-4-Bora-3a,4a-Diaza-s-Indacene-3-Dodecanoic Acid) (ThermoFisher) was injected into 3 dpf zebrafish larvae to observe lipid metabolism and transport by fluorescence. The compound was diluted in canola oil to facilitate transport (42) and concentrations of roughly 1 ng/μl injected. Imaging was performed using AxioZoom V16 (Zeiss) for overview images and close ups with LSM780 (Zeiss). Fluorescence intensity was measured using ImageJ. At 3dpf, BODIPY ratio was calculated by measuring average intensity in the yolk and the mean of the average intensities of dorsal aorta and posterior caudal vein. At 5 dpf, Ratio was calculated by measuring average intensity in the yolk and swim bladder area (matched with the brightfield image to determine area).

### Statistical analysis

All experiments have been performed for a minimum of three biological replicates from different crossings (N) and “n” represents the number of larvae used per replicate. Significance was determined using either Student’s *t* test or one-way ANOVA. All statistics were determined with Prism-GraphPad® (GraphPad). Significance was presented with the following ∗<0.05; ∗∗<0.01, and ∗∗∗<0.001.

## Results

### Misexpression of lipid metabolism enzymes in *chd7*^*−/−*^ mutants

**Fig. 1:**
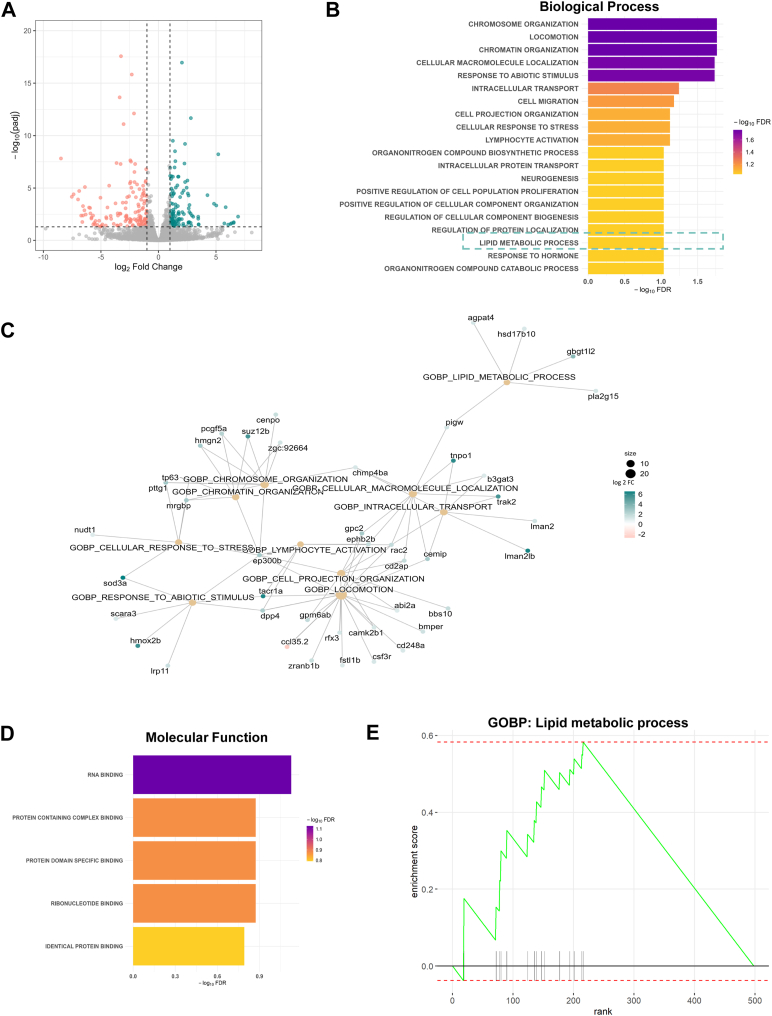
RNA-Seq Analysis of *chd7*^*−/−*^ mutants. A: Volcano plot showing transcriptomic responses (each gene plotted according to its log2 fold change) in 2 dpf whole zebrafish *chd7*^*−/−*^ embryos, relative to *chd7*^*+/+*^ controls (N = 3 from 10 pooled fish per sample/genotype). Differentially expressed genes (*P* < 0.05) are in salmon (downregulated) or teal (upregulated). B: Biological processes that are enriched in the differentially expressed genes. C: Cnet plot representing the linkage between the significantly dysregulated genes to enriched biological processes (BP) from gene set enrichment analysis in *chd7*^*−/−*^ mutants. D: Molecular function that are enriched in the differentially expressed genes. E. Enrichment plots from gene set enrichment analysis (GSEA) representing the top-enriched BP in *chd7*^*−/−*^ mutants. Each vertical black line marks the position in the ranked gene list of a gene that belongs to the BP:Lipid Metabolic Process gene set. The green curve represents the running enrichment score. The peak (maxima) of the green line is the enrichment score that is reported for this gene set. The red dashed lines show the maximum and minimum values of the running enrichment score.

To gain insights into molecular alterations in developing zebrafish embryos upon loss of chd*7* function, we performed high-throughput RNA-Seq analysis on embryonic 48 hpf *chd7*^*−/−*^ mutants and *chd7*^*+/+*^ controls. Following DEseq2 analysis, we identified 210 and 165 genes that were significantly downregulated and upregulated, respectively, in *chd7*^*−/−*^ mutants (Fig. 1A). We then explored the functional annotation of different genes in 48 hpf *chd7*^*−/−*^ mutants using Gene Ontology (GO) enrichment analysis and GSEA analysis. The DEGs were significantly involved in biological process (GO: BP), including chromatin organisation, locomotion and lipid metabolic process (Fig. 1B, C). The DEGs were enriched in the molecular (GO: MF) category and included RNA binding, and protein binding (Fig. 1D). The GSEA on DEGs revealed the enrichment of lipid metabolic process, indicating a significant dysregulation in the expression of lipid metabolism-related genes upon chd7 deficiency (Fig. 1E). This finding prompted us to investigate the potential dysregulated genes involved in lipid metabolism process in older 5 dpf fish from our published RNA sequence data (GSE139623; (17). Interestingly, in 5-day-old larval zebrafish, the GSEA analysis on DEGs also revealed the enrichment of lipid metabolic process (supplemental Fig. S1A, B). In the DEGs list, we also identified an enrichment of a set of 10 genes (*elovl2*, *hsd17b7*, *pla1a*, *fads2*, *fabp11b*, *pltp*, *osbpl1a*, *srebf2*, *soat2*, *fabp7b*) involved in lipid metabolism (supplemental Fig. S1C). Additionally, several genes can be clustered in potential pathways such as PPAR signaling pathway (*fabp7b*, *fabp11b*, *fads2*) and fatty acid metabolism (*elovl2*, *fads2*) (supplemental Fig. S1C).

#### Fatty acid and lipid metabolism are disturbed in chd7^−/−^ mutants

**Fig. 2:**
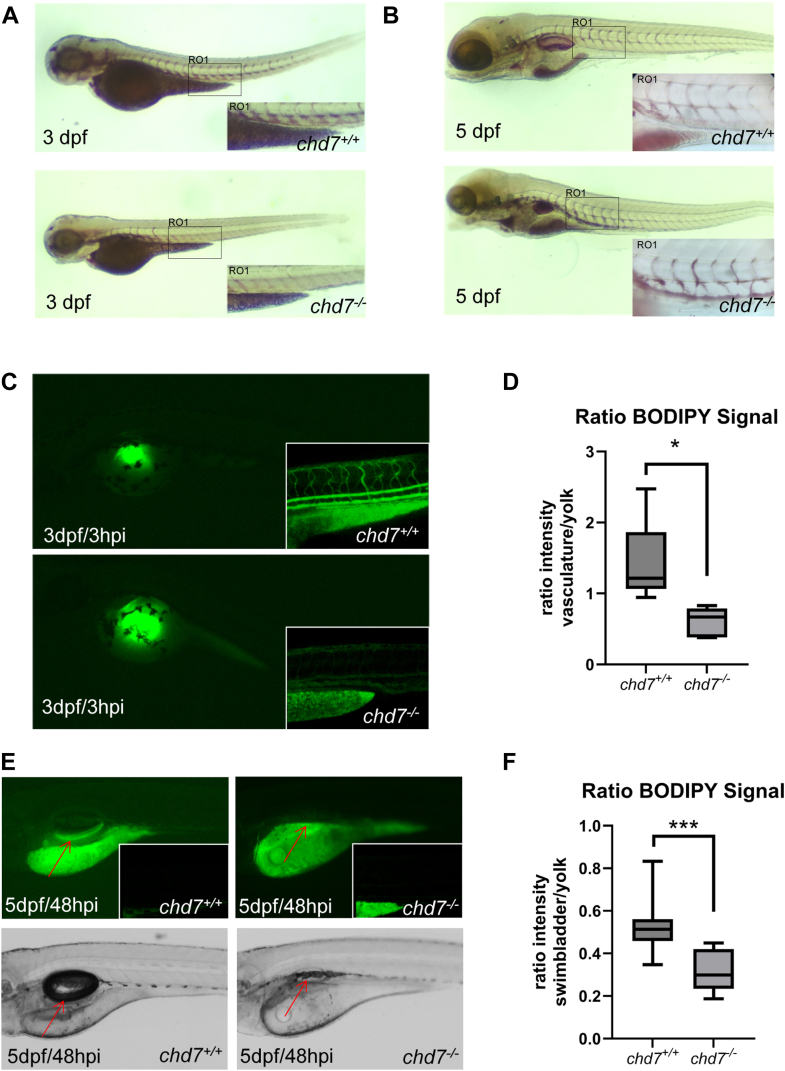
Lipid dyslipidemia and transport in *chd7*^*+/+*^ and *chd7*^*−/−*^ mutants. A: Representative images for 3 independent experiments (n = 15 larvae per replicate and genotype) of neutral lipid ORO staining in 3 dpf *chd7*^*+/+*^ (top) and *chd7*^*−/−*^ mutants (bottom). B: Representative images for 3 independent experiments (N) of ORO staining in 5 dpf *chd7*^*+/+*^ (top) and *chd7*^*−/−*^ mutants (bottom) with close up of caudal vein region (*chd7*^*+/+*^; N = 3, n = 12 and *chd7*^*−/−*^; N = 3, n = 11). C: Representative imaging for 3 independent experiments of BODIPY FL-C12 fluorescence at 3 dpf and 3 hpi *chd7*^*+/+*^ (n = 6 larvae per experiment) and *chd7*^*−/−*^ mutants (n = 5 larvae per experiment) with overview and close up of vascular system. D: Quantitative analysis of BODIPY signal. Ratio of average intensity measured in the yolk against intensity in the vasculature. (N = 3, n = 5 larvae per experiment). E: Imaging of BODIPY FL-C12 fluorescence at 5 dpf and 48 hpi *chd7*^*+/+*^ (n = 5 larvae per experiment) and *chd7*^*−/−*^ mutants (n = 4 larvae per experiment) and close up of vascular system. Representative images for 3 independent experiments (n represents larvae per sample/genotype). F: Quantitative analysis of BODIPY signal. Ratio of fluorescence intensity measured in the yolk against swim bladder. (N = 3, n = 4–5 larvae per experiment). (Significance: ∗*P* < 0.05; ∗∗*P* < 0.01; ∗∗∗*P* < 0.001, Error bars represent SD). Images were taken using a Zeiss Apotome.

To understand the effect of dysregulation of key lipid metabolism genes we determined the distribution of lipids in the developing larvae by staining for neutral lipids using Oil-Red-O (ORO). Controls showed distributed neutral lipids in the yolk and in the vascular system at 3 dpf. We observed that *chd7*^*−/−*^ mutants present with a more accumulated staining in yolk and less distribution in the vascular system (Fig. 2A). In *chd7*^*+/+*^ larvae, at 5 dpf, neutral lipid staining is also observed evenly distributed in the vasculature, swim bladder and the heart. However, in *chd7*^*−/−*^ mutants, a more accumulated staining in the swim bladder and uneven distribution in the vascular system was observed. This depletion was also noticeable towards the posterior of the larvae (Fig. 2B). Strikingly, in our ORO staining, we observed major accumulation of neutral lipids and lipid droplets in the caudal vein of *chd7*^*−/−*^ mutants (Fig. 2B, RO1).

### Fatty acid transport from yolk to embryo is impaired

To further investigate if the changed lipid distribution, previously observed in the ORO staining is caused by altered lipid transport and/or metabolism, we injected fluorescently labelled fatty acid chains (BIODIPY FL-C12) and observed their transport from yolk to vascular system and swim bladder mesothelium. Both *chd7*^*+/+*^ and *chd7*^*−/−*^ mutants show rapid diffusion of fluorescence hours after injections inside the yolk (Fig. 2C). Already after 3 h post injection (hpi) fluorescence can be seen in the vascular system in both *chd7*^*+/+*^ and *chd7*^*−/−*^ mutants (Fig. 2C close up). Nonetheless, fluorescence is significantly lower in *chd7*^*−/−*^ mutants (Fig. 2D). At 24 hpi, *chd7*^*+/+*^ show fluorescence in the entire yolk, intestinal tract, vascular system and near the swim bladder. In *chd7*^*−/−*^ mutants, fluorescence is observed in yolk and vascular system (supplemental Fig. S2A); however, no signal can be seen at the swim bladder (arrows). Notably, there appears to be greater accumulation in the intestinal tract. By 48 hpi and 5 dpf, the fluorescence in controls is distributed effectively throughout the larvae in the vascular system, intestinal tract and swim bladder (Fig. 2E). However, *chd7*^*−/−*^ mutants have significantly lower BODIPY signal at the swim bladder compared to the injected yolk (Fig. 2E arrows, 2F). Notably, we observed high excretion of the fluorescent lipid by the intestinal tract and resulting overall reduced level of fluorescence retained in the larvae (Fig. 2E). Altogether, the data suggest that lipid transport is significantly affected in *chd7*^*−/−*^ mutants.

### chd7^−/−^ mutants accumulate lipid droplets and fail to inflate swim bladder

**Fig. 3:**
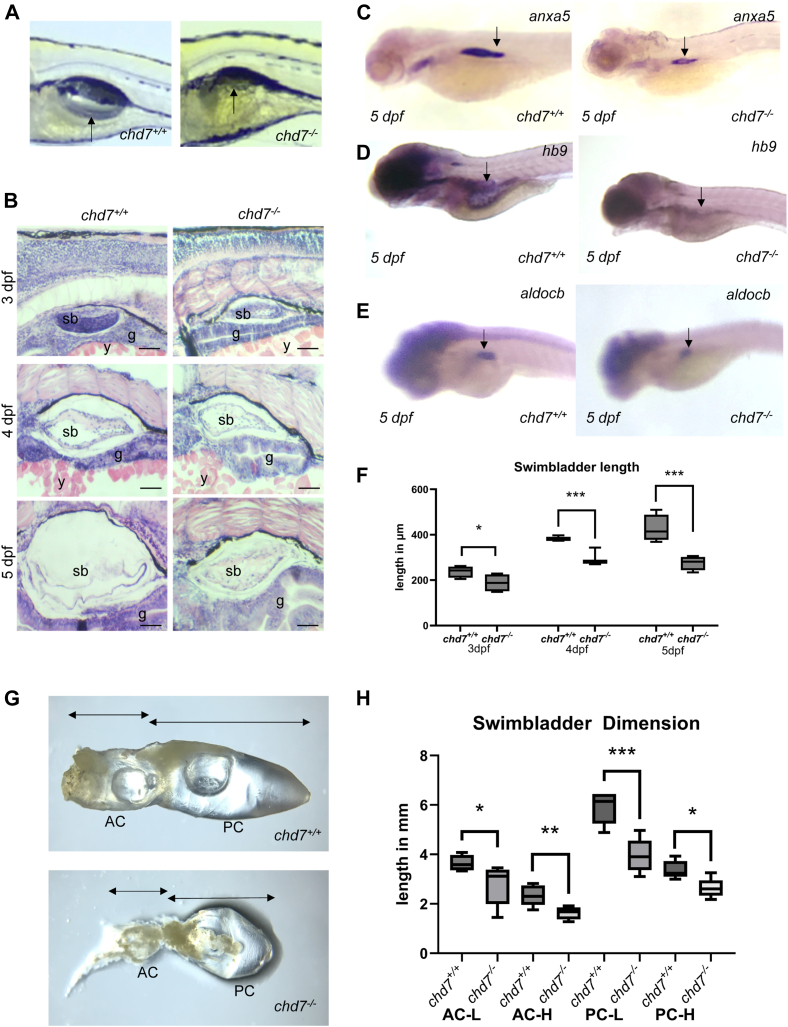
Swim bladder development in *chd7*^*−/−*^ mutants. A: Representative images for 3 independent experiments (n = 12 larvae per genotype) of swim bladder development in *chd7*^*+/+*^ and *chd7*^*−/−*^ mutants at 5 dpf showing failure to inflate the swim bladder in mutants (arrows). B: HE staining of swim bladder development in *chd7*^*+/+*^ and *chd7*^*−/−*^ mutants between 3 and 5 dpf showing generation of the three layers (outer mesothelium, mesenchyme and epithelium). Tissues are labelled g = gut, sb = swim bladder, y = yolk. Scale Bar 50 μm. Representative images for 3 independent experiments (n = 6 larvae per sample/genotype). C: Whole mount in situ hybridization for outer mesothelium marker *anxa5* expressed in the swim bladder (arrows) (3 experiments (N = 3), n = 20 larvae for each experiment). D: Whole mount in situ hybridization for epithelium marker *hb9* expressed in the swim bladder (arrows) (N = 3, n = 20 larvae for each experiment). E: Whole mount in situ hybridization for mesenchyme marker *aldocb* expressed in the swim bladder (arrows) (N = 3, n = 20 larvae for each experiment). F: Swim bladder length based on *anxa5* WISH (supplemental Fig. S2E) in 3–5 dpf old larvae (N = 3, n = 10 for each experiment). G: Representative adult swim bladder in *chd7*^*+/+*^ and *chd7*^*−/−*^ mutants with regions indicated of anterior chamber (AC) and posterior chamber (PC) of the swim bladder. H: Adult swim bladder dimensions, indicating significant smaller anterior and posterior chambers in *chd7*^*−/−*^ mutants (*chd7*^*+/+*^ N = 6 and *chd7*^*−/−*^ N = 5) (AC: anterior chamber, PC: posterior chamber; H:height; L:length) (Significance: ∗*P* < 0.05; ∗∗*P* < 0.01; ∗∗∗*P* < 0.001, Error bars represent SD).

Morphological evaluation showed that *chd7*^*−/−*^ mutants fail to inflate the swim bladder by 5 dpf (Fig. 3A). Observation of *chd7*^*−/−*^ mutants revealed that 80% of mutants present with this phenotype, which does not recover over an extended time (up to 8 dpf), while *chd7*^*+/+*^ larvae show a properly inflated swim bladder by 4–5 dpf (supplemental Fig. S2B). H&E staining revealed a successful formation of the swim bladder by budding from the gut and separation of the three major layers of epithelium, mesenchymal layer and outer mesothelium at 3 dpf, in both control and *chd7*^*−/−*^ mutants (Fig. 3B). However, from 4 dpf we noticed halted elongation and failure to inflate afterwards. By 5 dpf we also observed a significantly decreased size of the swim bladder in comparison to *chd7*^*+/+*^, yet all three layers remain present as identified by the expression of markers (outer mesothelium (*anxa5*), epithelium (*hb9*) and mesenchyme (*aldocb*)) for each layer (Fig. 3C–E). We used *anxa5* to determine size of the swim bladder based on the outer mesothelium and identified a significantly smaller size from 3 dpf to 5 dpf (Fig. 3F and supplemental Fig. S2E). We found no difference in proliferation in the developing swim bladder layers (supplemental Fig. S4A, B). At 5 dpf *chd7*^*−/−*^ larvae were significantly smaller in size than wild-type larvae, consistent with our previous reports and growth retardation as a common feature of CHARGE syndrome (Supplemental Fig. S2C; (15, 17). Notably, the low percentage of fish that do inflate the swim bladder are more likely to survive. Nonetheless, analysis of adult swim bladder shows that *chd7*^*−/−*^ mutants are still present with a significantly smaller swim bladder of both anterior (AC) and posterior chamber (PC) (Fig. 3G, H), suggesting that the swim bladder defect is likely not solely attributable to an early developmental delay.

### Outer mesothelium markers and swim bladder-specific lipid metabolism enzymes are depleted

**Fig. 4:**
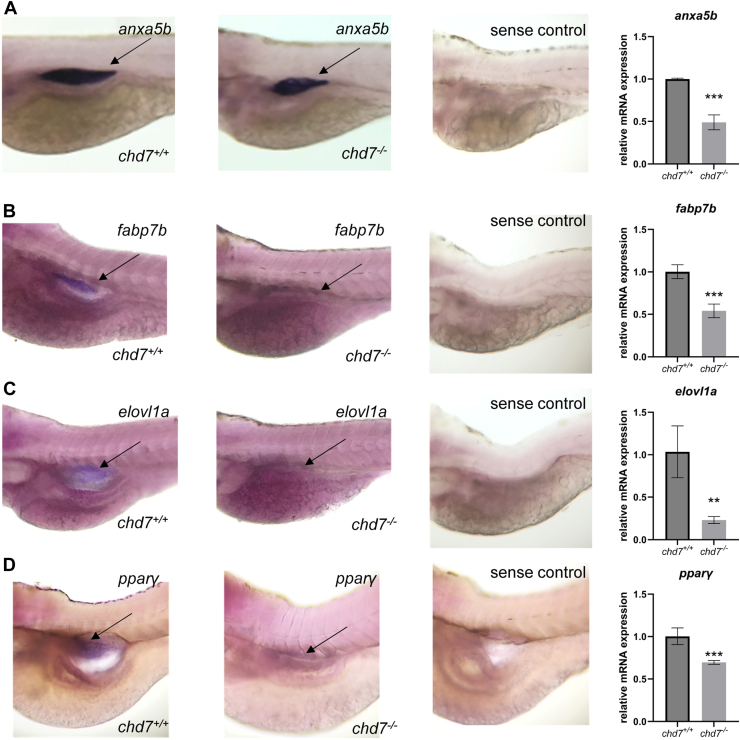
Pparγ regulated lipid metabolism. A–D: Representative images for WISH of 5dpf *chd7*^*+/+*^ and *chd7*^*−/−*^ larvae, including sense control, as well as results for RT-qPCR of key lipid genes in the swim bladder showing significant reduction for *anxa5*, *fabp7b*, *elovl1* and *pparγ* at 5dpf. WISH (N = 3, n = 18–20 larvae for each staining) and RT-qPCR (N = 4 from 15 pooled fish per sample/genotype). Larvae for each experiment were taken from age-matched *chd7*^*+/+*^ or *chd7*^*−/−*^ independent clutches (N = 3–4; significance: ∗*P* < 0.05; ∗∗*P* < 0.01; ∗∗∗*P* < 0.001; Error bars represent SD).

Investigations into the early swim bladder development revealed no effect on budding and layer formation. Yet, we observed a striking depletion of mesothelium markers post 3 dpf in *chd7*^*−/−*^ mutants. While at 3 dpf the marker *anxa5*, efficiently expressed in the outer mesothelium and linked to fatty acid metabolism, remains unchanged, at 5 dpf the expression is significantly depleted by more than two-fold in both qPCR and WISH (Fig. 4A). Furthermore, the fatty acid binding protein 7b, *fabp7b*, is also depleted in the outer mesothelium and expression levels (Fig. 4B). Finally, the expression of fatty acid hydrolase, *elovl1a*, also expressed in outer mesothelium, is almost completely lost at 5 dpf (Fig. 4C). The swim bladder-associated genes are significantly downregulated in qPCR analysis at both the onset of inflation (5 dpf) (Fig. 4A–C) and remain downregulated post-inflation period (9 dpf) (supplemental Fig. S3B).

### Lipid metabolism is only partially regulated by pparγ in chd7^−/−^ mutants

The Peroxisome Proliferator-Activated Receptor gamma (PPARγ) is a master regulator of lipid metabolism and transport (43). PPARγ cooperates with the transcription factor CCAAT-enhancer binding proteins (C/EBPs) to regulate lipid metabolism, and CHD7 can directly regulate CEBPA (the gene encoding C/EBPα) and CEBPB (the gene encoding C/EBPβ) through chromatin remodeling and direct promoter binding (44, 45). Interestingly, *pparγ* is significantly downregulated in *chd7*^*−/−*^ zebrafish mutants (Fig. 4D) along with the CEBP homologs *cebpa* and *cebpb* (supplemental Fig. S3A).

In zebrafish, *ppar*γ expression was not detected at 1 dpf by WISH (46). From 2 dpf on, *ppar*γ expression was detected in developing zebrafish, with a strong *ppar*γ expression in the liver, the gut, and the swim bladder by 5 dpf (46). We thus sought to examine the expression of *ppar*γ around the developing gut and swim bladder area in *chd7*^*−/−*^ zebrafish mutants. Consistent with our qPCR data, we observed that *pparγ* expression is significantly downregulated in *chd7*^*−/−*^ mutants, including in the swim bladder at 5dpf (Fig. 4D) and even before the onset of inflation (3 dpf; supplemental Fig. S2D) and after post-inflation periods (9 dpf; supplemental Fig. S3B).

**Fig. 5:**
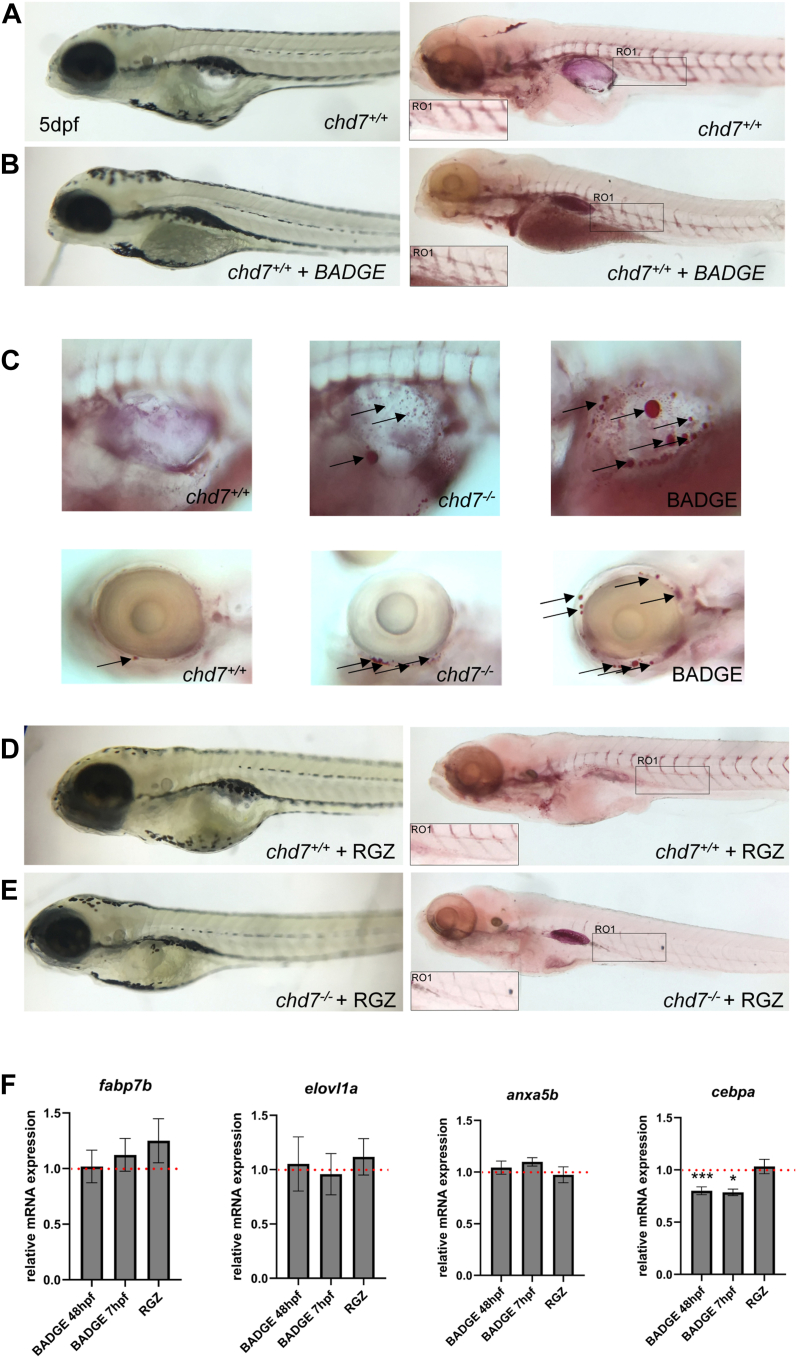
PPARγ in swim bladder development. A: Representative morphology and ORO staining of sibling 5dpf *chd7*^*+/+*^ (vehicle treated) and B: BADGE treated larvae with close up of caudal vein region C: ORO staining showing neutral lipid droplet accumulation in the eye vasculature and swim bladder (arrows) of 5dpf *chd7*^*+/+*^(vehicle treated), *chd7*^*−/−*^ mutants and BADGE treated *chd7*^*+/+*^ larvae. D: Representative morphology and ORO staining of 5dpf *chd7*^*+/+*^ and (E) *chd7*^*−/−*^ mutants larvae treated with RGZ, with a close-up of the caudal vein region. A–E: representative images for 3 independent experiments (n = 5larvae per condition and genotype). F. RT-qPCR of key swim bladder lipid metabolism markers *fabp7b, elov1a, anxa5b* and *cebpa* in *chd7*^*+/+*^ (vehicle-treated), early and late onset BADGE-treated and RGZ-treated larvae. RT-qPCR were performed with sibling wildtype (*chd7*^*+/+*^) larvae of 3 separate clutches (N) (N = 3 from 15 pooled fish per sample/genotype; significance: ∗*P* < 0.05; ∗∗*P* < 0.01; ∗∗∗*P* < 0.001; Error bars represent SD).

We next tried to determine if Pparγ dependent lipid metabolism is the underlying effector of the observed phenotype in *chd7*^*−/−*^ fish. We used a Pparγ specific inhibitor, BADGE, previously used in zebrafish to study lipid metabolism, to analyze the same lipid metabolism markers with an early (48 hpf) and late onset (72 hpf) inhibition as to not interfere with swim bladder budding (46). *chd7*^*−/−*^ mutants led to a reduced ORO stain throughout the body. We also observed large lipid droplets in the swim bladder and eye vasculature, specifically the circumferential vein, upon BADGE treatment (Fig. 5C arrows). BADGE treated larvae also showed failure to inflate the swim bladder and ORO staining showed striking similarity to *chd7*^*−/−*^ mutants with reduced overall neutral lipids and accumulation of lipid droplets in the caudal vein and swim bladder (Fig. 5A, B). Inhibition and severity of effect could be shown in a dose-dependent manner for BADGE (supplemental Fig. S5). BADGE treatment also reduced the expression of *cebpa* (Fig. 5F). These data demonstrate that Pparγ signaling inhibitor with BADGE phenocopies features observed in *chd7*^*−/−*^ mutant fish.

We next sought to investigate whether treatment with a Pparγ agonist, Rosiglitazone (RGZ), which was previously used in zebrafish (47), could ameliorate lipid metabolism related phenotypes in *chd7*^*−/−*^ mutants. Vascular system phenotypes in *chd7*^*−/−*^ mutants treated with RGZ showed reduced accumulation of neutral lipids (Fig. 5D, E). Additionally, the treatment improves overall lipid distribution in *chd7*^*−/−*^ mutants (Fig. 5E) However, swim bladder inflation and lipid droplet accumulation remained unaffected (Fig. 5E).

**Fig. 6:**
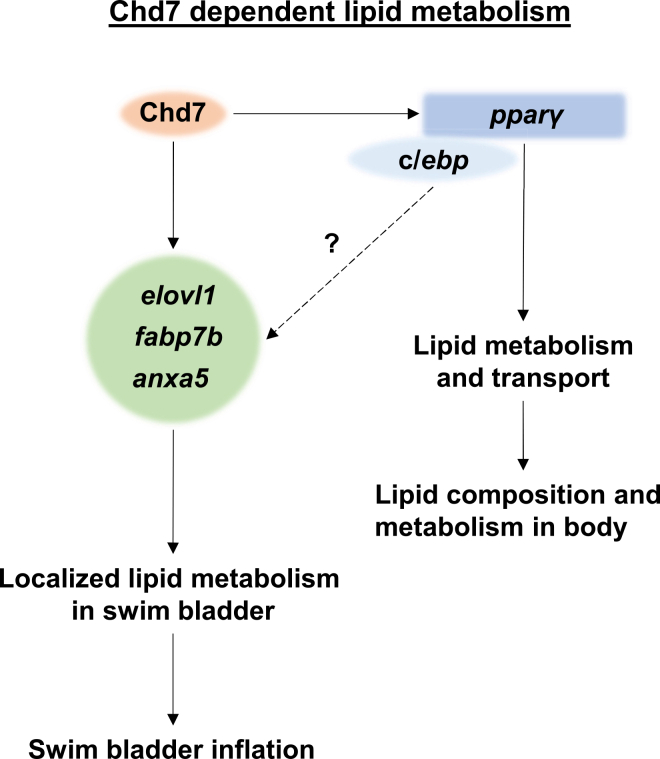
Schematic representation of the proposed pathways that would result in the observed phenotype showing lipid accumulation specifically to the swim bladder based on partial Pparγ activity.

Further testing for gene expression data, we could show that the swim bladder specific lipid enzymes of this study, *anxa5*, *elovl*1 and *fabp7b* remained unaffected by Pparγ targeting by both antagonist and agonist (Fig. 5F). Furthermore, there was no difference if the inhibition/activation was performed prior to swim bladder elongation (48 hpf) or afterward (72 hpf) (Fig. 5F). The lack of response in the expression underlines a specific regulation of Chd7 on swim bladder lipid metabolism genes separate from the known regulation via Pparγ (Fig. 6). Analysis of the chromatin immunoprecipitation-sequencing (ChIP-seq) datasets from the ENCODE Transcription Factor Targets project (48) confirmed that dysregulated genes *anxa5, elovl*1 and *fabp7* are direct targets of CHD7 in murine and human cell lines (supplemental Fig. S6). Altogether, these data suggest that dyslipidemia in *chd7*^*−/−*^ mutants is likely occurring via two independent Chd7 pathways (Fig. 6).

## Discussion

Chd7 has been shown to have a high regulatory impact on neural crest development, stem cell maintenance, GABAergic networks, craniofacial development and spinal development, among others (6, 7, 11, 15, 17). Here, we show that during larval development, chd7 deficiency results in dyslipidemia, with accumulation of lipid droplets and subsequent failure to inflate the swim bladder.

### Chd7 regulates lipid metabolism

RNA Seq analysis at 48 hpf and 5 dpf shows the striking regulatory role of Chd7 for lipid metabolism-related genes. Abnormal lipid metabolism has previously been observed in CHD7-deficient oligodendrocytes and mesenchymal stem cells (3, 11). Furthermore, Chd7 is known to directly regulate Pparγ, which acts as a master lipid regulator (49). Pparγ in zebrafish has been shown to regulate adipose tissue development located below the swim bladder (50). Our study could indicate that this lipid accumulation may initially be required to supply the swim bladder layers and allow for sufficient plasticity and ability to inflate the swim bladder.

### Lipid metabolism and swim bladder development

It was described earlier that lipids are known to be particularly important for swim bladder development and function. Marine fish show varying lipid composition and some even dependent on a lipid filled bladder to control buoyancy (32, 51, 52). Our analysis with ORO, suggests an altered lipid distribution and transport in the developing larvae. Quantification of lipid transport from the yolk to the vasculature of the embryo, as shown by BODIPY FL-C12 injections, reveal a lower rate of transport early on. At 5dpf, the lipid integration of BOIDPY FL-C12 into the swim bladder is reduced, but not lost, possibly supporting that it is not fully functionally affected and may suggest a separate uptake or metabolism pathway for the swim bladder in which the misregulated genes of this study are highly involved in. Previous studies have shown that mutants, such as “fat free,” which have deficient phospholipid and cholesterol metabolism also present with a deflated swim bladder (53). In line with our data, that shows a significant depletion in the key lipid metabolic enzyme elovl1, a knockdown model for *elovl1* causes dyslipidemia, neutral lipid accumulation and failure to inflate the swim bladder (28). This may indicate that the loss of *elovl1* expression in *chd7*^*−/−*^ mutants is at least in part the causative factor. The consequential impairment in long chain fatty acid synthesis, such as DHA, in the swim bladder locality, may be specifically relevant for the proper lipid composition and metabolism. ELOVL1^−/−^ mice have shown that ELOVL1 function is required to produce sufficient levels of ceramides and proper fatty acid chain length, to guarantee epidermal barrier function such as permeability (54). Permeability and elasticity are key functional requirements for swim bladder tissue layers. ELOVL1^−/−^ mice also showed low survival rate postnatally and displayed neural deficits, such as decreased myelin and reduced acoustic startle response (55). Additionally, the significant reduction of *fabp7b*, which has specific binding affinity to DHA, may be a targeted function for the swim bladder, but may also show implications for brain development (56). *FABP7*^*−/−*^ mice have also shown that FABP7 deficiency may have implications in retinal vasculature maintenance (57). Furthermore, FABP7 is required for neural stem cell maintenance, oligodendrocyte differentiation and myelination, which are functions also previously linked to CHD7 (3, 4, 58, 59, 60).

While our data show that global inhibition of Pparγ is sufficient to mimic the lipid phenotype, gene expression suggests that *elovl1*, *fabp7b* and *anxa5* expression are independent of Pparγ activity. In fact, all dysregulated genes, including *pparγ*, *cebp*, *fabp7b*, *anxa5*, and *elovl1* have been previously shown to be direct targets of Chd7 (48). Therefore, dyslipidemia in *chd7*^*−/−*^ mutants is likely occurring via two independent Chd7 pathways (Fig. 6).

The occurrence of large lipid droplets within the swim bladder of *chd7*^*−/−*^ mutants further supports the hypothesis of an inefficient metabolizing of lipids. Noticeably, this striking characteristic of failure of swim bladder inflation appears in correlation with survival of homozygous mutants (17).

Although *chd7*^*−/−*^ larvae display significant growth retardation and developmental delay at 5 dpf (supplemental Fig. S2C), consistent with previous reports and with growth retardation as a common feature of CHARGE syndrome, we cannot exclude that this generalized delay influences multiple developmental pathways and may contribute to the swim bladder phenotypes we observe. Nonetheless, we provide several lines of evidence that indicate that the swim bladder defect in *chd7*^*−/−*^ fish is not solely attributable to an early developmental delay. First, developmental delay often shows recoverable, proportional phenotypes across organs, yet adult *chd7*^−/−^ fish still exhibit a significantly smaller anterior and posterior swim-bladder chamber (Fig. 3G, H). Second, in situ hybridization and RT-qPCR demonstrate persistent downregulation of *pparγ* (and other key lipid regulators) in the swim bladder territory itself at 3, 5, and 9 dpf, time points during and after the window of overt developmental delay. Third, developmental delay in the swim bladder at early developmental stage in zebrafish is typically a consequence of reduced proliferation during the growth phase (27, 31, 61). We observed no difference in proliferation in the developing swim bladder layers between 5 dpf *chd7*^−/−^ and *chd7*^+/+^ larvae (supplemental Fig. S4A, B). Additionally, we observed a successful formation of the swim bladder by budding from the gut and separation of the three major layers of epithelium, mesenchymal layer and outer mesothelium at the same developmental 3 dpf stage, in both control and *chd7*^−/−^ mutants (Fig. 3B). These findings suggest that Chd7 deficiency exerts a more direct, tissue-specific effect on *pparγ* expression and swim bladder morphogenesis that is superimposed on the broader growth retardation caused by CHD7 loss. Future swim bladder specific and temporal rescue and/or knockout experiments will be required to disentangle cell-autonomous versus non-autonomous contributions of Chd7 to these processes.

### Neural implications

Previously published RNA-Seq data (17), detected dysregulated expression for lipid metabolism in the larval brain. The overlap between genes in whole 48 hpf zebrafish and 5 dpf brain indicate a possible requirement of function in both neural and other tissue development. The importance of proper lipid metabolism for neural survival and neural stem cell regulation has been previously addressed, including regulation by PPARα (62, 63, 64, 65). In fact, Chd7 has previously been associated with lipid metabolism required in oligodendrocytes and myelin production, which was depleted and affected neural development and myelination (3, 8). In line with all these studies, we could observe significant downregulation of myelin and oligodendrocyte markers *plp1b* and *mbp* in our *chd7*^*−/−*^ mutants (supplemental Fig. S4C). Future studies will show if we can target lipid metabolism to ameliorate neural deficits in CS.

### Lipid metabolism and patients with CS

CHARGE syndrome patients have not been characterized with dyslipidemias, to the best of our knowledge. Lipid screens are not commonly reported for patients, considering the wide range of phenotypes and if lipid profiles are reported, they were within the accepted range (66). However, these profiles report LDL, HDL and triglycerides exclusively. Therefore, specific fatty acid depletions or accumulations may be overlooked in these analyses. A more detailed analysis of DHA, PUFAs or long-chain fatty acids is not known to us. It would be of great interest to see if future investigations in patients can determine if this dyslipidemia is conserved.

In conclusion, our study identifies a regulatory deficit of lipid metabolism controlled by Chd7 in the developing zebrafish, partially via Pparγ. Furthermore, it is the first study to show the regulation of lipid metabolism in the early swim bladder, which is dependent on gene regulation of *elovl1* and *fabp7b* by Chd7 and independent of Pparγ.

## Data availability

All data supporting the conclusions of this article are contained within the article and will be made available to other researchers upon reasonable request.

## Supplemental data

This article contains supplemental data.

## Author contributions

G. F., M. B., C. Z., P. J., and B. K. methodology; G. F., M. B., P. J., B. K., and J. S. formal analysis; M. B. and S. P. writing – review & editing; M. B. writing – original draft; M. B. and S. P. supervision; M. B. data curation; M. B. and S. P. conceptualization. S. P. funding acquisition; S. C. investigation.

## Conflict of interest

The authors declare that they have no conflicts of interest with the contents of this article.

## References

[1] Feng W., Shao C., Liu H.K. (2017). Versatile roles of the chromatin remodeler CHD7 during brain development and disease. Front. Mol. Neurosci..

[2] Bajpai R., Chen D.A., Rada-Iglesias A., Zhang J., Xiong Y., Helms J. (2010). CHD7 cooperates with PBAF to control multipotent neural crest formation. Nature.

[3] He D., Marie C., Zhao C., Kim B., Wang J., Deng Y. (2016). Chd7 cooperates with Sox10 and regulates the onset of CNS myelination and remyelination. Nat. Neurosci..

[4] Jones K.M., Saric N., Russell J.P., Andoniadou C.L., Scambler P.J., Basson M.A. (2015). CHD7 maintains neural stem cell quiescence and prevents premature stem cell depletion in the adult hippocampus. Stem Cells.

[5] Marie C., Clavairoly A., Frah M., Hmidan H., Yan J., Zhao C. (2018). Oligodendrocyte precursor survival and differentiation requires chromatin remodeling by Chd7 and Chd8. Proc. Natl. Acad. Sci. U. S. A..

[6] Ohta S., Yaguchi T., Okuno H., Chneiweiss H., Kawakami Y., Okano H. (2016). CHD7 promotes proliferation of neural stem cells mediated by MIF. Mol. Brain.

[7] Okuno H., Renault Mihara F., Ohta S., Fukuda K., Kurosawa K., Akamatsu W. (2017). CHARGE syndrome modeling using patient-iPSCs reveals defective migration of neural crest cells harboring CHD7 mutations. Elife.

[8] Shi L., Wang Z., Li Y., Song Z., Yin W., Hu B. (2023). Deletion of the *chd7* hinders oligodendrocyte progenitor cell development and myelination in zebrafish. Int. J. Mol. Sci..

[9] Schnetz M.P., Handoko L., Akhtar-Zaidi B., Bartels C.F., Pereira C.F., Fisher A.G. (2010). CHD7 targets active gene enhancer elements to modulate ES cell-specific gene expression. PLoS Genet..

[10] Whittaker D.E., Riegman K.L., Kasah S., Mohan C., Yu T., Pijuan-Sala B. (2017). The chromatin remodeling factor CHD7 controls cerebellar development by regulating reelin expression. J. Clin. Invest..

[11] Liu C., Xiong Q., Li Q. (2022). CHD7 regulates bone-fat balance by suppressing PPAR-γ signaling. Nat. Commun..

[12] Janssen N., Bergman J.E., Swertz M.A., Tranebjaerg L., Lodahl M., Schoots J. (2012). Mutation update on the CHD7 gene involved in CHARGE syndrome. Hum. Mutat..

[13] Pagon R.A., Graham J.M., Zonana J., Yong S.L. (1981). Coloboma, congenital heart disease, and choanal atresia with multiple anomalies: CHARGE association. J. Pediatr..

[14] Zentner G.E., Layman W.S., Martin D.M., Scacheri P.C. (2010). Molecular and phenotypic aspects of CHD7 mutation in CHARGE syndrome. Am. J. Med. Genet. A..

[15] Breuer M., Rummler M., Singh J., Maher S., Zaouter C., Jamadagni P. (2024). CHD7 regulates craniofacial cartilage development via controlling HTR2B expression. J. Bone Mineral Res..

[16] Cloney K., Steele S.L., Stoyek M.R., Croll R.P., Smith F.M., Prykhozhij S.V. (2018). Etiology and functional validation of gastrointestinal motility dysfunction in a zebrafish model of CHARGE syndrome. FEBS J..

[17] Jamadagni P., Breuer M., Schmeisser K., Cardinal T., Kassa B., Parker J.A. (2021). Chromatin remodeller CHD7 is required for GABAergic neuron development by promoting PAQR3 expression. EMBO Rep..

[18] Liu Z.Z., Wang Z.L., Choi T.I., Huang W.T., Wang H.T., Han Y.Y. (2018). Chd7 is critical for early T-Cell development and thymus organogenesis in zebrafish. Am. J. Pathol..

[19] Patten S.A., Jacobs-McDaniels N.L., Zaouter C., Drapeau P., Albertson R.C., Moldovan F. (2012). Role of Chd7 in zebrafish: a model for CHARGE syndrome. PLoS One.

[20] Perry S.F., Wilson R.J., Straus C., Harris M.B., Remmers J.E. (2001). Which came first, the lung or the breath?. Comp. Biochem. Physiol. A, Mol. Integr. Physiol..

[21] Zheng W., Wang Z., Collins J.E., Andrews R.M., Stemple D., Gong Z. (2011). Comparative transcriptome analyses indicate molecular homology of zebrafish swim bladder and mammalian lung. PLoS One.

[22] Hsu P., Ma A., Wilson M., Williams G., Curotta J., Munns C.F. (2014). CHARGE syndrome: a review. J. Paediatr. Child Health.

[23] Sporik R., Dinwiddie R., Wallis C. (1997). Lung involvement in the multisystem syndrome CHARGE association. Eur. Respir. J..

[24] Cass A.N., Servetnick M.D., McCune A.R. (2013). Expression of a lung developmental cassette in the adult and developing zebrafish swim bladder. Evol. Dev..

[25] Lee G.H., Cheng N.W., Yu H.H., Tsai J.N., Liu T., Wen Z.H. (2019). A novel zebrafish model to emulate lung injury by folate deficiency-induced swim bladder defectiveness and protease/antiprotease expression imbalance. Sci. Rep..

[26] Zhang Y., Liu H., Yao J., Huang Y., Qin S., Sun Z. (2016). Manipulating the air-filled zebrafish swim bladder as a neutrophilic inflammation model for acute lung injury. Cell Death Dis..

[27] Winata C.L., Korzh S., Kondrychyn I., Zheng W., Korzh V., Gong Z. (2009). Development of zebrafish swim bladder: the requirement of Hedgehog signaling in specification and organization of the three tissue layers. Dev. Biol..

[28] Bhandari S., Lee J.N., Kim Y.I., Nam I.K., Kim S.J., Kim S.J. (2016). The fatty acid chain elongase, Elovl1, is required for kidney and swim bladder development during zebrafish embryogenesis. Organogenesis.

[29] Chen Y., Wang M., Chen D., Wang J., Kang N. (2016). Chromatin remodeling enzyme CHD7 is necessary for osteogenesis of human mesenchymal stem cells. Biochem. Biophys. Res. Commun..

[30] Winata C.L., Korzh S., Kondrychyn I., Korzh V., Gong Z. (2010). The role of vasculature and blood circulation in zebrafish swim bladder development. BMC Dev. Biol..

[31] Yin A., Korzh S., Winata C.L., Korzh V., Gong Z. (2011). Wnt signaling is required for early development of zebrafish swim bladder. PLoS One.

[32] Daniels C.B., Skinner C.H. (1994). The composition and function of surface-active lipids in the goldfish swim bladder. Physiol. Zoolog..

[33] Anderson J.L., Carten J.D., Farber S.A. (2011). Zebrafish lipid metabolism: from mediating early patterning to the metabolism of dietary fat and cholesterol. Methods Cell Biol..

[34] Zeituni E.M., Farber S.A. (2016). Studying lipid metabolism and transport during zebrafish development. Methods Mol. Biol. (Clifton, N.J.).

[35] Jaya-Ram A., Kuah M.-K., Lim P.-S., Kolkovski S., Shu-Chien A.C. (2008). Influence of dietary HUFA levels on reproductive performance, tissue fatty acid profile and desaturase and elongase mRNAs expression in female zebrafish Danio rerio. Aquaculture.

[36] Watanabe T. (1993). Importance of docosahexaenoic acid in marine larval fish. J. World Aquaculture Soc..

[37] Matsunari H., Hashimoto H., Oda K., Masuda Y., Imaizumi H., Teruya K. (2013). Effects of docosahexaenoic acid on growth, survival and swim bladder inflation of larval amberjack. (Seriola dumerili, Risso).

[38] Westerfield M. (1993).

[39] Kimmel C.B., Ballard W.W., Kimmel S.R., Ullmann B., Schilling T.F. (1995). Stages of embryonic development of the zebrafish. Dev. Dyn..

[40] Thisse C., Thisse B. (2008). High-resolution in situ hybridization to whole-mount zebrafish embryos. Nat. Protoc..

[41] Schlegel A., Stainier D.Y. (2006). Microsomal triglyceride transfer protein is required for yolk lipid utilization and absorption of dietary lipids in zebrafish larvae. Biochemistry.

[42] Miyares R.L., de Rezende V.B., Farber S.A. (2014). Zebrafish yolk lipid processing: a tractable tool for the study of vertebrate lipid transport and metabolism. Dis. Model Mech..

[43] Ahmadian M., Suh J.M., Hah N., Liddle C., Atkins A.R., Downes M. (2013). PPARγ signaling and metabolism: the good, the bad and the future. Nat. Med..

[44] Chai M., Sanosaka T., Okuno H., Zhou Z., Koya I., Banno S. (2018). Chromatin remodeler CHD7 regulates the stem cell identity of human neural progenitors. Genes Dev..

[45] Schulz Y., Wehner P., Opitz L., Salinas-Riester G., Bongers E.M., van Ravenswaaij-Arts C.M. (2014). CHD7, the gene mutated in CHARGE syndrome, regulates genes involved in neural crest cell guidance. Hum. Genet..

[46] Fraher D., Sanigorski A., Mellett N.A., Meikle P.J., Sinclair A.J., Gibert Y. (2016). Zebrafish embryonic lipidomic analysis reveals that the yolk cell is metabolically active in processing lipid. Cell Rep..

[47] Venezia O., Islam S., Cho C., Timme-Laragy A.R., Sant K.E. (2021). Modulation of PPAR signaling disrupts pancreas development in the zebrafish, Danio rerio. Toxicol. Appl. Pharmacol..

[48] Rouillard A.D., Gundersen G.W., Fernandez N.F., Wang Z., Monteiro C.D., McDermott M.G. (2016). The harmonizome: a collection of processed datasets gathered to serve and mine knowledge about genes and proteins. Database (Oxford).

[49] Takada I., Kouzmenko A.P., Kato S. (2009). Wnt and PPARgamma signaling in osteoblastogenesis and adipogenesis. Nat. Rev. Rheumatol..

[50] Den Broeder M.J., Kopylova V.A., Kamminga L.M., Legler J. (2015). Zebrafish as a model to study the role of peroxisome proliferating-activated receptors in adipogenesis and obesity. PPAR Res..

[51] Neighbors M.A., Nafpaktitis B.G. (1982). Lipid compositions, water contents, swim bladder morphologies and buoyancies of nineteen species of midwater fishes (18 myctophids and 1 neoscopelid). Mar. Biol..

[52] Patton S., Thomas A.J. (1971). Composition of lipid foams from swim bladders of two deep ocean fish species. J. Lipid Res..

[53] Farber S.A., Pack M., Ho S.-Y., Johnson I.D., Wagner D.S., Dosch R. (2001). Genetic analysis of digestive physiology using fluorescent phospholipid reporters. Science.

[54] Sassa T., Ohno Y., Suzuki S., Nomura T., Nishioka C., Kashiwagi T. (2013). Impaired epidermal permeability barrier in mice lacking elovl1, the gene responsible for very-long-chain fatty acid production. Mol. Cell Biol..

[55] Isokawa M., Sassa T., Hattori S., Miyakawa T., Kihara A. (2019). Reduced chain length in myelin sphingolipids and poorer motor coordination in mice deficient in the fatty acid elongase Elovl1. FASEB Bioadv..

[56] Liu R.-Z., Denovan-Wright E.M., Degrave A., Thisse C., Thisse B., Wright J.M. (2004). Differential expression of duplicated genes for brain-type fatty acid-binding proteins (fabp7a and fabp7b) during early development of the CNS in zebrafish (Danio rerio). Gene Expr. Patterns.

[57] Su X., Tan Q.S.W., Parikh B.H., Tan A., Mehta M.N., Sia Wey Y. (2016). Characterization of fatty acid binding protein 7 (FABP7) in the Murine Retina. Invest. Ophthalmol. Vis. Sci..

[58] Foerster S., Guzman de la Fuente A., Kagawa Y., Bartels T., Owada Y., Franklin R.J.M. (2020). The fatty acid binding protein FABP7 is required for optimal oligodendrocyte differentiation during myelination but not during remyelination.

[59] Matsumata M., Sakayori N., Maekawa M., Owada Y., Yoshikawa T., Osumi N. (2012). The effects of Fabp7 and Fabp5 on postnatal hippocampal neurogenesis in the mouse. Stem Cells.

[60] Sharifi K., Ebrahimi M., Kagawa Y., Islam A., Tuerxun T., Yasumoto Y. (2013). Differential expression and regulatory roles of FABP5 and FABP7 in oligodendrocyte lineage cells. Cell Tissue Res..

[61] Yin A., Korzh V., Gong Z. (2012). Perturbation of zebrafish swimbladder development by enhancing Wnt signaling in Wif1 morphants. Biochim. Biophys. Acta.

[62] Hamilton L.K., Fernandes K.J.L. (2018). Neural stem cells and adult brain fatty acid metabolism: lessons from the 3xTg model of Alzheimer's disease. Biol. Cell.

[63] Knobloch M. (2017). The role of lipid metabolism for neural stem cell regulation. Brain Plasticity (Amsterdam, Netherlands).

[64] Pearsall E.A., Cheng R., Zhou K., Takahashi Y., Matlock H.G., Vadvalkar S.S. (2017). PPARα is essential for retinal lipid metabolism and neuronal survival. BMC Biol..

[65] Rodríguez-Berdini L., Caputto B.L. (2019). Lipid metabolism in neurons: a brief story of a novel c-Fos-Dependent mechanism for the regulation of their synthesis. Front. Cell. Neurosci..

[66] Chakraborty S., Chakraborty J. (2012). CHARGE Association. Indian J. Endocrinol. Metab..

