# Supplementary material for: Chd7 regulates lipid metabolism and swim bladder inflation in zebrafish
Source: J Lipid Res. 2026 May 18;67(6):101062. doi: 10.1016/j.jlr.2026.101062 (PMC13311276; doi:10.1016/j.jlr.2026.101062)
Supplement: Supplemental Figures [file mmc1.pdf]

Supplementals Fig S1

A

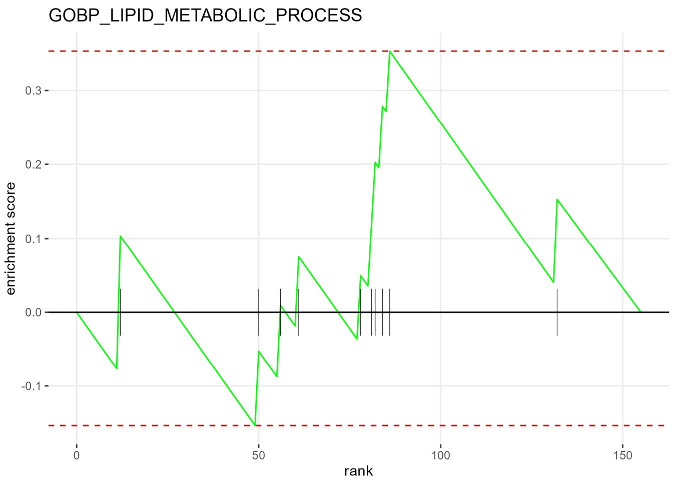

B

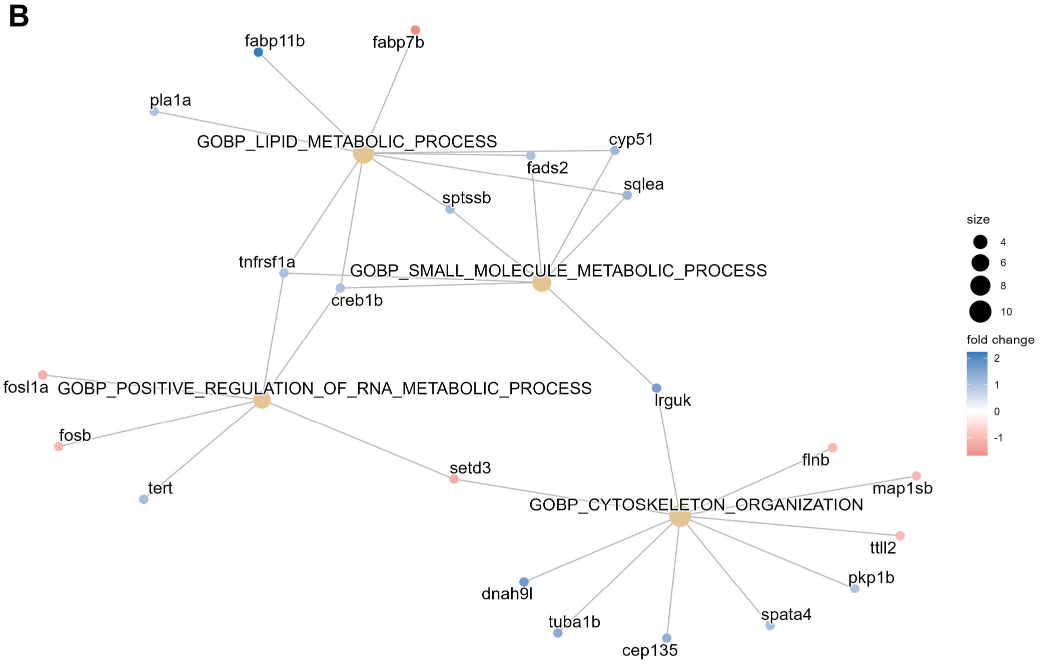

C

Lipid metabolic process (GO:0006629)			
Gene	*FC*	*padj*	
elovl2	*1.67*	*0.00289*	
hsd17b7	*1.52*	*0.03159*	
pla1a	*2.02*	*7.3E-07*	
fads2	*2.06*	*4.8E-08*	
fabp11b	*4.68*	*7.6E-29*	
pltp	*-1.62*	*0.00114*	
osbpl1a	*-1.49*	*0.00289*	
srebf2	*1.27*	*0.02716*	
soat2	*-1.63*	*0.00915*	
fabp7b	*-3.21*	*3E-16*	

Supp Fig S1. Lipid metabolism dysregulation in 5-day-old chd7-/- mutants. A. Enrichment plots from gene set enrichment analysis (GSEA) representing top enriched BP in 5 dpf chd7-/- mutants. Each vertical black line marks the position in the ranked gene list of a gene that belongs to the BP:Lipid Metabolic Process gene set. The green curve represents the running enrichment score. The peak (maxima) of the green line is the enrichment score that is reported for this gene set. The red dashed lines show the maximum and minimum values of the running enrichment score. B. CNET plot representing the linkage between the significantly dysregulated genes to enriched biological processes (BP) from gene set enrichment analysis in *chd7$^{-/-}$* mutants. C. List of enriched dysregulated genes in 5 dpf zebrafish that are involved in lipid metabolism.

Supplementals Fig S2

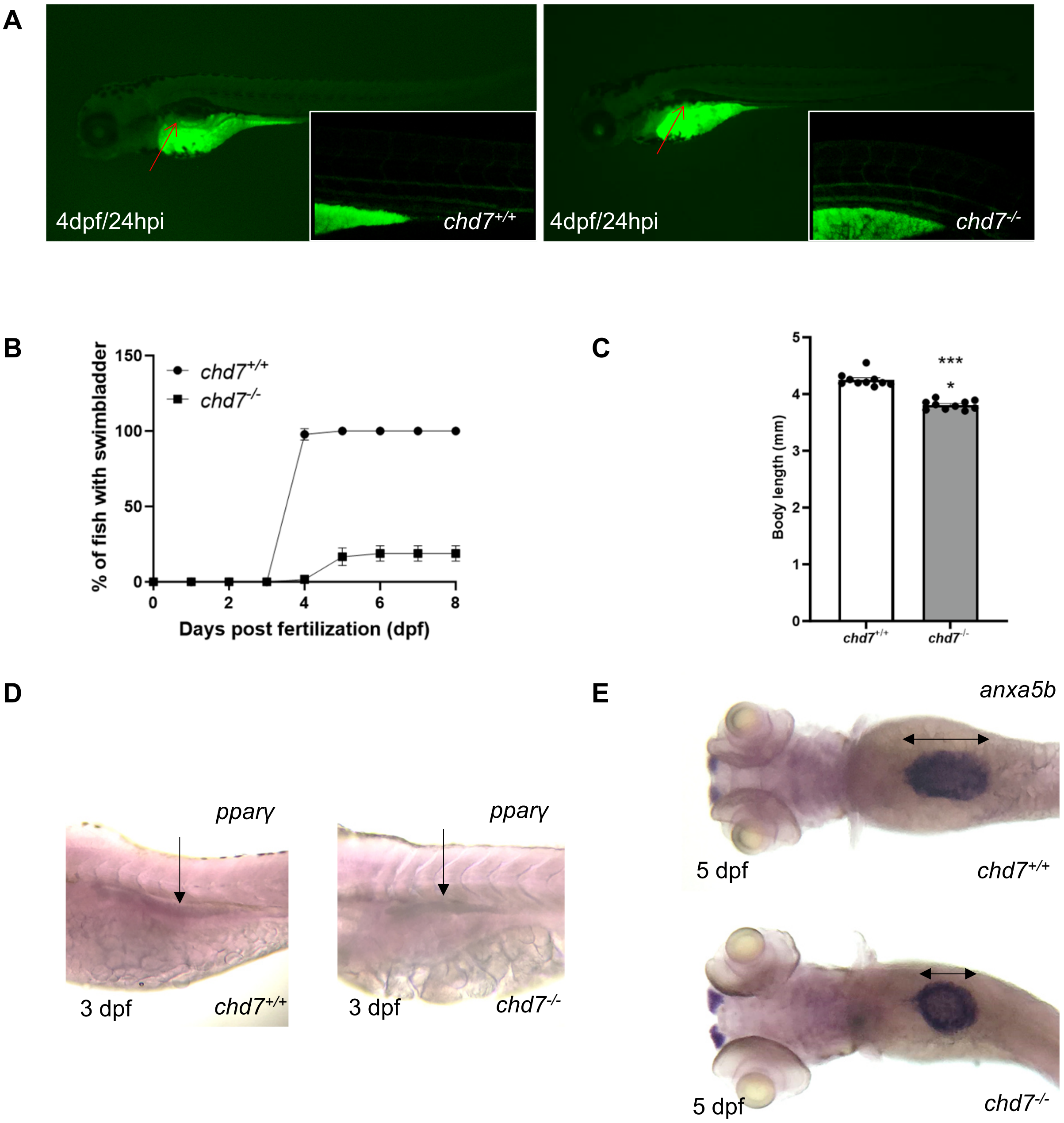

Supp Fig S2. A. Imaging of BODIPY FL-C12 fluorescence at 4 dpf and 24 hpi *chd7*$^{+/+}$ (n=6 larvae per experiment) and *chd7*$^{-/-}$ mutants (n=5 larvae per experiment) and close up of vascular system. Representative images for 3 independent experiments. B. Swim bladder inflation in *chd7*$^{+/+}$ and *chd7*$^{-/-}$ zebrafish over time indicating notably delayed and decreased number of inflation in mutants (N=3, n=30) C. Body length of 5dpf *chd7*$^{+/+}$ and *chd7*$^{-/-}$. (N=3, n=10) D. Representative WISH for *pparg* at 3dpf in *chd7*$^{+/+}$ and *chd7*$^{-/-}$ (N=3, n=20 larvae for each experiment). E. Dorsal view of 5 dpf *anxa5* WISH (left) in *chd7*$^{+/+}$ and *chd7*$^{-/-}$ used for swim bladder measurements (arrows) (N=3, n=10 for each experiment) (Significance: *p<0.05; **p<0.01; ***p<0.001, Error bars represent SD)

Supplementals Fig S3

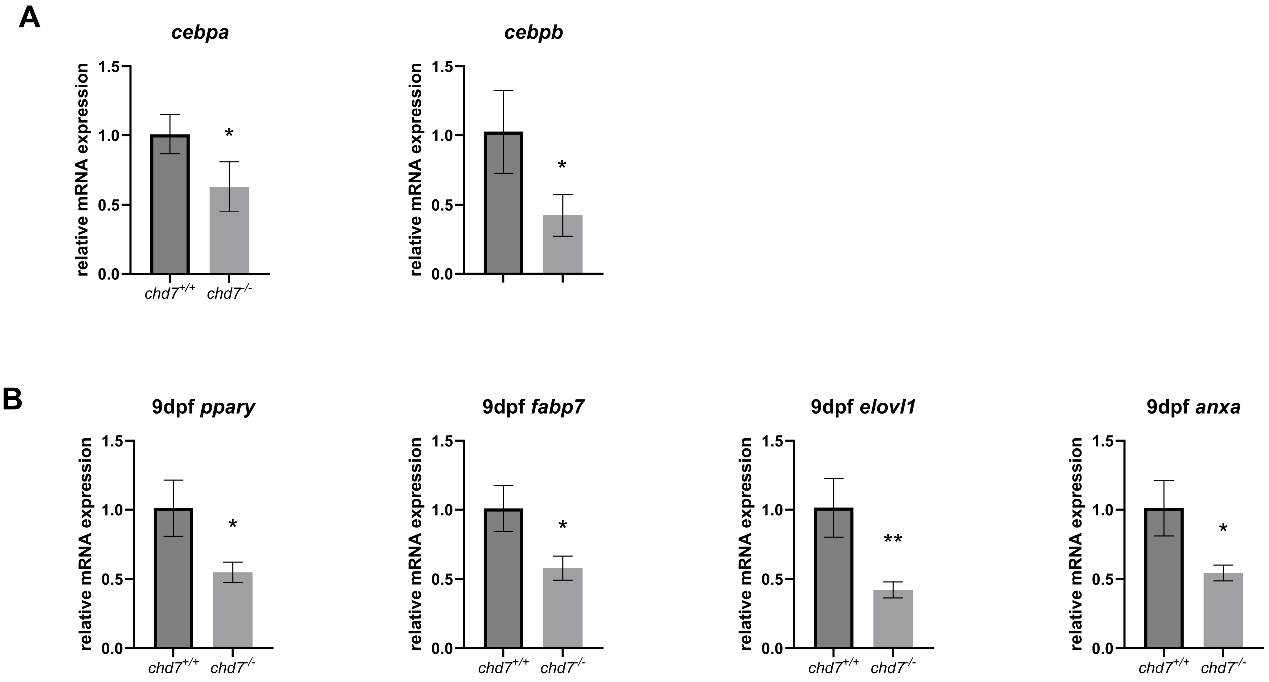

Supp Fig S3. A. RT-qPCR of key lipid metabolism enzymes *cebpa* and *cebpb* at 5dpf and B. *ppary, fabp7b, elovl1a* and *anxa5b* at 9dpf (N=3-4 from 15 pooled fish per sample/genotype) (Significance: *p<0.05; **p<0.01; ***p<0.001, Error bars represent SD)

Supplementals Fig S4

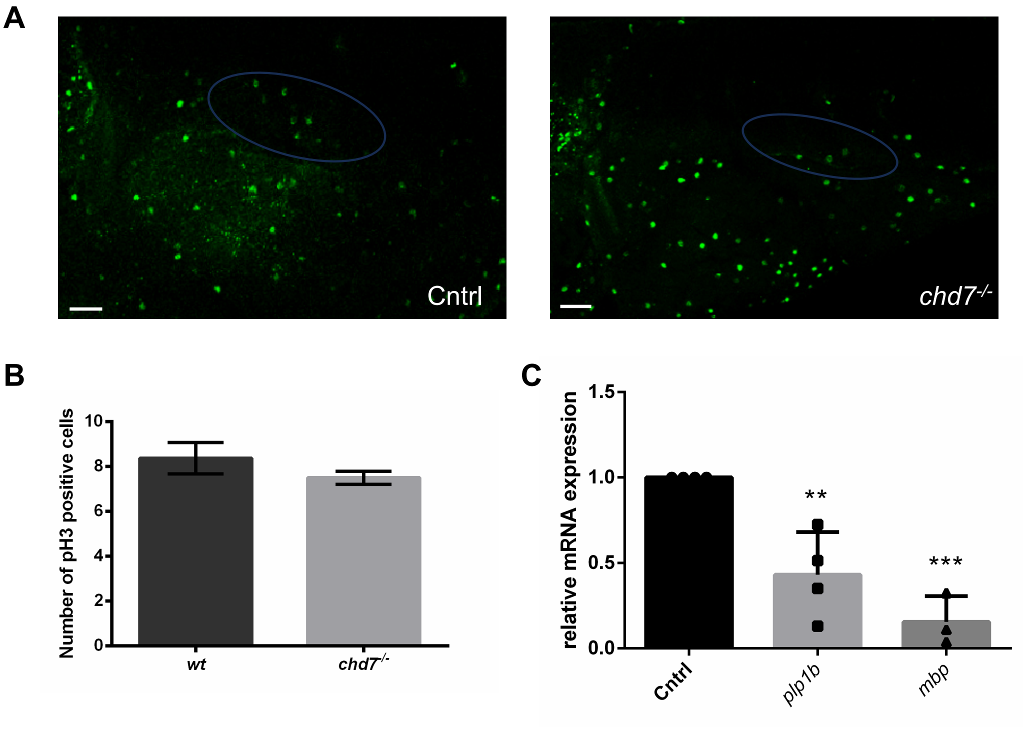

Supp Fig S4. A. pH3 positive cells in 5dpf Cntrl and *chd7*$^{-/-}$ zebrafish B. showing no significant differences (n=6 for each) C. RT-qPCR of myelin and oligondedrocyte markers *plp1b* and *mbp* in 5dpf *chd7*$^{+/+}$ and *chd7*$^{-/-}$ zebrafish (N=4,from 15 pooled fish per sample/genotype) (Significance: *p<0.05; **p<0.01; ***p<0.001, Error bars represent SD)

Supplementals Fig S5

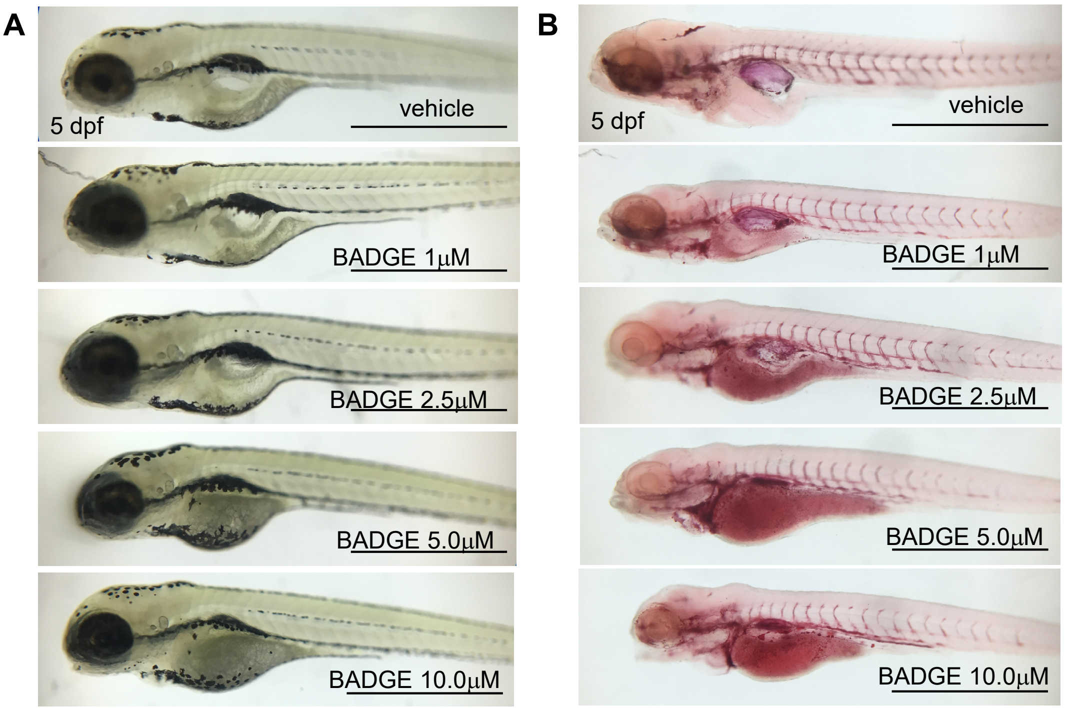

Supp Fig S5 A. Representative morphology overview of 5dpf larvae treated with vehicle and increasing concentrations of BADGE (1 to 10µm) B. ORO staining of 5dpf larvae treated with vehicle and increasing concentrations of BADGE (1 to 10µm) showing an increased effect on neutral lipid distribution with increasing concentration. All 5dpf larvae were siblings from *chd7*$^{+/+}$ control crosses. Representative images for 3 independent experiments (n=5 larvae per condition and genotype).

Supplementals Fig S6

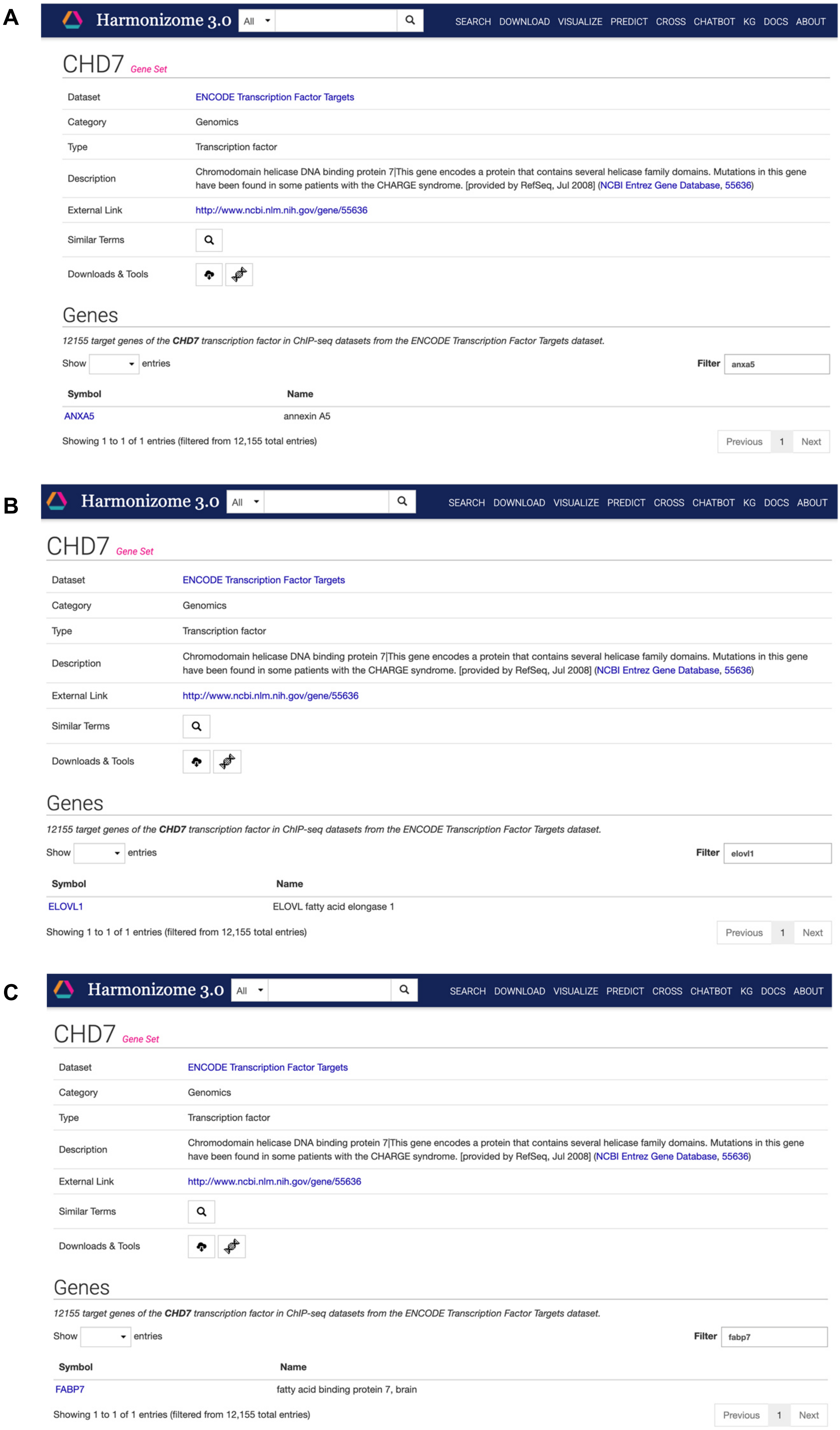

Supp Fig S6. Lipid metabolism genes *ANXA5, ELOVL1 and FABP7* are direct targets of CHD7. Analysis of target genes of transcription factor CHD7 in ChIP-seq datasets from the ENCODE Transcription Factor Targets dataset revealed *ANXA5*(A), *ELOVL1*(B)and *FABP7*(C) as direct targets of CHD7.

Supplementals

Supp Table 1. Primer list used for RT-qPCR and WISH probe generation

	Target gene	Forward Primer	Reverse Primer
RT-qPCR	*elf1α*	GTGGCTGGAGACAGCAAGA	AGAGATCTGACCAGGGTGGTT
	anxa5B	TGATACCGGGGGACATTTCG	ACAGAGCCTGAGCATCACTTT
	elovl1a	TGACGCACGATTGATCAGGT	CGCACTCTGGCATCAGTTCT
	fabp7b	ACCGACACTGCAAATCCACT	ACGTCCCCAAAGGTGAGTTT
	pparγ	CCGAGTGTGTGGAGACAAAGC	CTTGTTGCGGCTCTTCTTGTG
	c/ebpa	TCGTCCGAACGCGACTCTAT	ACCGAAATTCTGACAGCAACA
	c/ebpb	ACTTGCGAAGTTCTCAGGGG	GCTGTGGAAAGCGAGGTAGT
	plp1b	CCTCTCTGGAGTGAGCGAAC	GCAGCAGTCATAGCAACCTAGA
WISH Primers	*anxa5b*	TGCAAAGCATGATGCCGAAG	CCCGCCTGTACGAGTATCAC
	fabp7b	TGGATGCTTTTTGTGGCACG	GTCCATTCTTTCAATGAGTTGGATG
	elovl1a	TGGCTGAAGAAACAACGACA	GGTGAATGAAGATGGGAACT
	pparg	GCCTGAAGCTGGTGTACGAT	TCGGCAGATCTGGACTGGTA
